# The Role of Chronic Liver Diseases in the Emergence and Recurrence of Hepatocellular Carcinoma: An Omics Perspective

**DOI:** 10.3389/fmed.2022.888850

**Published:** 2022-06-24

**Authors:** Sofia Zanotti, Gina F. Boot, Mairene Coto-Llerena, John Gallon, Gabriel F. Hess, Savas D. Soysal, Otto Kollmar, Charlotte K. Y. Ng, Salvatore Piscuoglio

**Affiliations:** ^1^Anatomic Pathology Unit, IRCCS Humanitas University Research Hospital, Milan, Italy; ^2^Visceral Surgery and Precision Medicine Research Laboratory, Department of Biomedicine, University of Basel, Basel, Switzerland; ^3^Institute of Medical Genetics and Pathology, University Hospital Basel, Basel, Switzerland; ^4^Clarunis, University Center for Gastrointestinal and Liver Diseases, St. Clara Hospital and University Hospital Basel, Basel, Switzerland; ^5^Department for BioMedical Research, University of Bern, Bern, Switzerland; ^6^Swiss Institute of Bioinformatics, Lausanne, Switzerland; ^7^Bern Center for Precision Medicine, Bern, Switzerland

**Keywords:** hepatocellular carcinoma, chronic liver disease, genomics, epigenetics, transcriptomics, proteomics, metabolomics

## Abstract

Hepatocellular carcinoma (HCC) typically develops from a background of cirrhosis resulting from chronic inflammation. This inflammation is frequently associated with chronic liver diseases (CLD). The advent of next generation sequencing has enabled extensive analyses of molecular aberrations in HCC. However, less attention has been directed to the chronically inflamed background of the liver, prior to HCC emergence and during recurrence following surgery. Hepatocytes within chronically inflamed liver tissues present highly activated inflammatory signaling pathways and accumulation of a complex mutational landscape. In this altered environment, cells may transform in a stepwise manner toward tumorigenesis. Similarly, the chronically inflamed environment which persists after resection may impact the timing of HCC recurrence. Advances in research are allowing an extensive epigenomic, transcriptomic and proteomic characterization of CLD which define the emergence of HCC or its recurrence. The amount of data generated will enable the understanding of oncogenic mechanisms in HCC from the CLD perspective and provide the possibility to identify robust biomarkers or novel therapeutic targets for the treatment of primary and recurrent HCC. Importantly, biomarkers defined by the analysis of CLD tissue may permit the early detection or prevention of HCC emergence and recurrence. In this review, we compile the current omics based evidence of the contribution of CLD tissues to the emergence and recurrence of HCC.

## Introduction

Despite the extensive characterization of the molecular landscape of hepatocellular carcinoma (HCC), the mechanisms through which the liver tissue drives the emergence and recurrence of HCC remain poorly understood. The most common etiologies of chronic liver disease (CLD) are chronic hepatitis B or C viral infection (HBV and HCV, respectively), alcohol-related liver disease (ALD), and non-alcoholic fatty liver disease (NAFLD) (1, 2). The unresolved inflammation associated with CLD often leads to fibrosis and subsequent cirrhosis, characterized by a buildup of scar tissue in the liver. Moreover, the resulting oxidative stress, through generation of reactive oxygen and nitrogen species in proliferating hepatocytes, as well as inflammatory cells responding to molecules released by damaged hepatocytes, promotes an environment that accelerates malignant transformation and survival of hepatocytes (3).

Despite surgical treatment in HCC yielding high survival outcomes at 5 years, the incidence of intrahepatic recurrence after curative resection remains unsatisfactory at 70% (1). Whereas, early recurrences (<2 years) typically arise from micrometastases following resection, late recurrences (>2 years) are consistent with a phenomenon known as the “field effect,” where *de novo* tumors arise due to an inflammatory carcinogenic microenvironment (4). In recent years, the focus of next generation sequencing profiling has shifted from delineating genomic biomarkers in tumoral tissue to acquiring a multi-omic overview of the tumor microenvironment (TME) and the characterization of the contribution of CLD tissue to HCC development. This review summarizes the current omics knowledge about HCC emergence and recurrence by investigating the putative involvement of CLD tissues.

## The Role of Chronic Liver Disease In HCC Emergence

### The Role of the Genetic Landscape in CLD in HCC Emergence

Several monogenic diseases trigger the development of cirrhosis and increase the risk for CLD-HCC initiation. These diseases are characterized by germline mutations of genes including *HFE1* (encoding MHC-I like molecules (5), *ATP7B* liver copper homeostasis (6), *FAH* [required during the tyrosine catabolic pathway (7), *UROD* essential for heme biosynthesis (8)], glucose-6-phosphatase (G6Pase) (9, 10) and *SERPINA1* [cofactor for TTR expression (11, 12)]. In addition, numerous single-nucleotide polymorphisms (SNPs) have been reported to be associated with HCC development (13). HCC-associated SNPs are not by themselves pathogenic *per se*, but require an additional cause of CLD. For example, the interplay between aflatoxin B1, HBV and SNPs of *GSTM1* and *GSTT1* (null alleles) increases the risk of HCC emergence (14). While SNP of *PNPLA3* (rs738409) is strongly associated with CLD (ALD and NAFLD) related HCC (15–17), its contribution is minor in HCC promoted by HCV infection (18, 19). Interestingly, in the context of hepatic fibrosis, cells overexpressing *PNPLA3* promoted immune cells chemotaxis (monocytes and macrophages) as compared to their wild type *PNPLA3* carriers (20). In addition to SNP of *PNPLA3* (rs738409), SNPs in *TM6SF2* (rs58542926), and *HSD17B13* (rs72613567) increase and decrease the risk of alcohol-related cirrhosis HCC, respectively (21). Whereas, SNPs in *WNT3A-WNT9A* (rs708113) were found to exert a protective role in hepatocarcinogenesis (22).

The higher mutational burden found in CLDs compared to the normal liver suggests positive selection shapes the genomic landscape during HCC emergence (23–27). Analysis of somatic mutations across healthy, ALD- and NAFLD-livers revealed that somatic mutations in *FOXO1, CIDEB*, and *GPAM* detected in CLD tissue showed convergent evolution. Of note, mutations in metabolism genes were observed in both ALD and NAFLD but less frequently in HCC (28). Ng et al. identified 7 significantly mutated genes across 122 HCC biopsies and showed 7% of samples harbored GPAM mutations (7 of 9 were frameshift mutations), which were more frequent than mutations in cancer-related genes such as *ARID2* and *RB1* (29). Mutations were identified in CLD tissues with allelic frequencies suggesting clonal expansion in CLD (26). Some non-driver mutations may become an advantageous asset for the liver's ability to not only withstand injuries but also to regenerate. In this study, genes involved in the control of liver regeneration were the most recurrently mutated within cirrhotic nodules, including driver mutations in cancer-related gene *ARID1A*, which controls gene expression associated with emergence of injury-induced liver progenitor-like cells (30). Recently, the relationship between *ARID1A* expression and immune cells that infiltrate tumors was clarified, whereby *ARID1A* expression in hepatocytes was positively associated with activated eosinophils, T helper cells, central memory T and Th2 cells (31). Frequent and cancer-promoting mutations in *ARID1A* and *TP53* were also acquired in subpopulations of hepatocytes in cirrhotic regenerating nodules, where an increase in clonal size was primarily observed in CLD but rarely in HCC cases (24). It is now well-established that alterations in *TP53* signaling pathways affect the TME at all stages of HCC development from initiation to metastasis, i.e., *via* hepatocytes, hepatic stellate cells (HSCs), immune cells, as well as cancer stem cells (32, 33). In one of the studies exploring the role of p53 in the immune response arising from embryonic liver progenitor cells, mutual *H-Ras* expression and *TP53* downregulation resulted in HCC development. The data indicated that p53 exerted a cytostatic effect by blocking proliferation and triggering cellular senescence, rendering cancer cells susceptible to recognition by the immune system (34). Cancer removal was affected by immune cells, including macrophages, neutrophils and NK cells (34–36). Non-hepatocyte tumor suppressive effects of *TP53* were mediated by its activity in HSCs. Lujambio et al. tested the conditional inactivation of p53 in HSCs during liver fibrosis. Depletion of p53 resulted in increased fibrosis and excessive extracellular matrix production, which eventually led to tumor occurrence and liver failure. It was shown that the formation of HCC did not directly evolve from p53-depleted HSCs, rather its deletion in these cells created a niche for hepatocytes malignant transformation (36). In addition, p53 expression levels (high or depleted) of HSCs were shown to modulate the different tumor-inhibiting phenotype of macrophages (M1 or M2, respectively) (36). It is important to stress that the previously mentioned experiments were performed in immunologically-compromised mice, thereby without an appropriate immune response. Nevertheless, these results support a model whereby physiological p53 activity in hepatocytes and HSCs facilitate tumor clearance. Liver injury also affects p53 in HSCs. For instance, following liver injury, the increase of p53 levels trigger senescence of HSCs, thus promoting clearance of the scar tissue by immune cells. This response can be viewed as a balancing factor between regeneration and malignant transformation: p53 signaling protects from transformation while activating progenitor cells for compensatory liver repopulation (36, 37).

**Figure 1:**
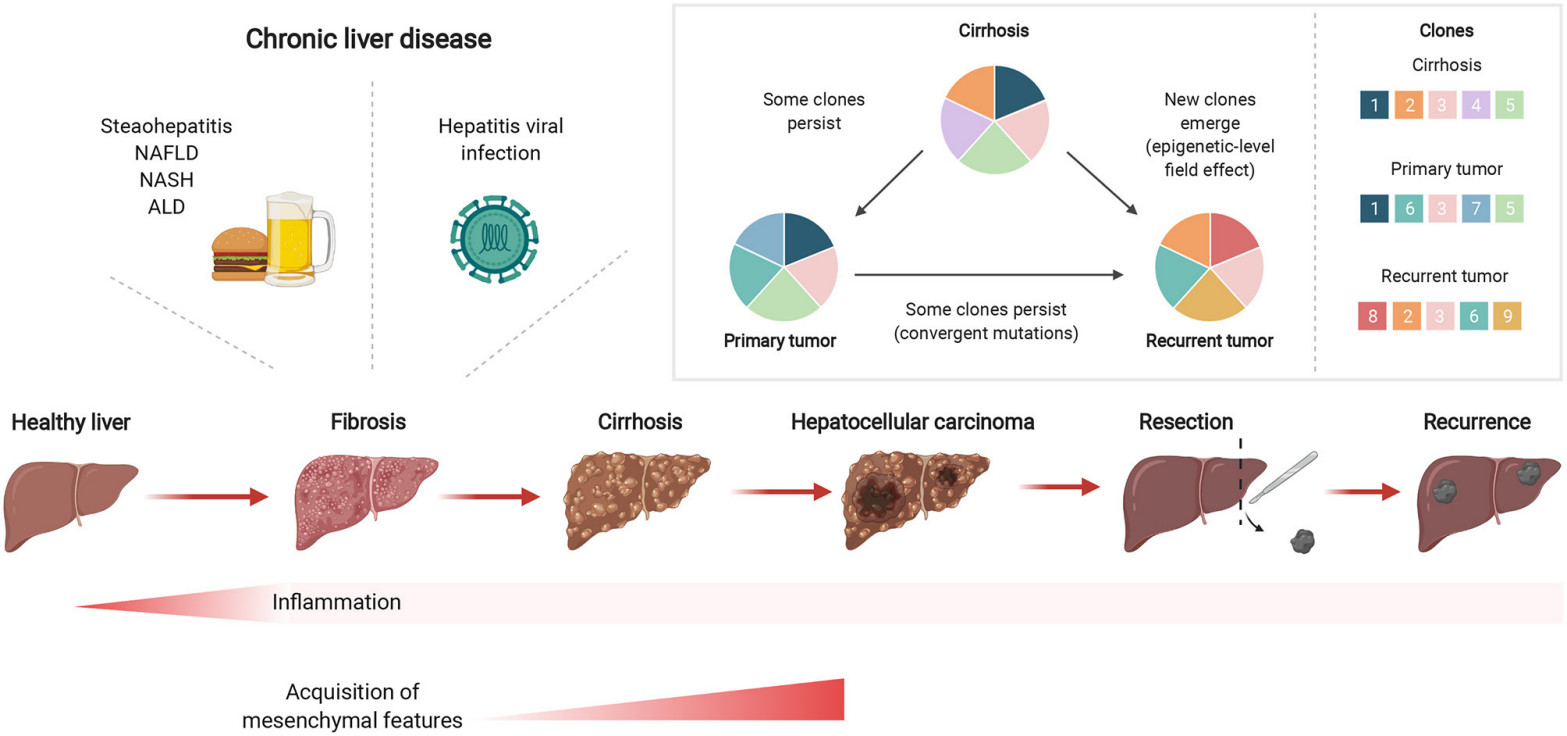
Chronic liver inflammation predisposes potential genetic changes for selective clonal expansion during HCC development. Inflammation triggered by chronic liver diseases, such as fatty liver disease or hepatitis viral infections, can eventually develop into fibrosis which can subsequently lead to cirrhosis. In cirrhotic tissues, hepatocarcinogenesis is a multistep process whereby the repeated cycles of cell damage and hepatocyte regeneration may predispose the accumulation of various multi-omic changes. Therefore, this chronic inflammatory microenvironment sets up a scenario that promotes the hepatocytes with an advantageous mutational burden for clonal expansion and eventually selects for HCC lesions. Created with www.BioRender.com.

Mutational signatures in cirrhotic liver are conserved in HCC (27), including aging-associated single base substitution and indel mutational signatures in CLD, relating to increased proliferation after tissue regeneration (38). Together with senescence, a state of permanent cell cycle arrest, short telomeres in hepatocytes are hallmarks of cirrhosis (39), where shortening occurs more quickly than through aging alone. *TERT* promoter mutations occur predominantly at two hotspots of chromosome 5 (40) and one of the key mechanisms involved in malignant transformation of cirrhotic hepatocytes is the reactivation of telomerase by mutations in its promoter such as the well-characterized −124 and −146 C>T transitions. In line with these observations, the *TERT* promoter is frequently mutated in premalignant cirrhotic nodules early during hepatocarcinogenesis (23). Recently, hotspot mutations in the *TERT* promoter were found to be altered in a focal nodular hyperplasia lesion and clonally related HCC lesions (41). In contrast to other studies, Kim et al. found that *TERT* promoter mutations were only detectable in HCC, yet not in the cirrhotic nodules (24). These findings suggest that *TERT* promoter mutations can frequently act as a gatekeeper event during the transformation sequence, but are not always necessary for HCC emergence. Importantly, mutations arising in regenerating nodules of CLD tissues suggest an early carcinogenic event depicting clonal expansion in the stepwise malignant transformation from CLD to HCC (Figure 1).

### Epigenetic Alterations Predispose Cells to Tumorigenesis

CLDs trigger mechanisms that lead not only to the accumulation of genetic mutations but also of various cooperative epigenetic changes. Epigenetic silencing plays a key role in HCC emergence, where promoter methylation of tumor suppressor genes such as *RASSF1A* are noted at high frequency during the progression from cirrhosis to HCC (42). Okamoto et al. reported that hepatitis infections trigger an innate immune response dependent on natural killer (NK) cells to elicit DNA methylation, including methylation of *RASSF1A* (43). Comparison of promoter methylation profiles in the CLD tissue from HCC patients (cirrhotic vs. non-cirrhotic) identified aberrant promoter methylation of *UGT1A7* and *PLG*, genes which function in fibrogenesis and detoxification of carcinogens, respectively (44). These results highlight the importance of elucidating the underlying mechanisms of CLD beyond the genomic level to better understand the evolution of HCC. In line with this, our group recently demonstrated that CLDs trigger the formation of an epigenetic niche that predisposes cells to oncogenic alteration (45). We developed a CLD-associated DNA methylation score with prognostic value in HCC. High CLD methylation score was associated with higher levels of *TP53* somatic alterations in HCC, a poor prognostic marker (46). Additionally, we showed that despite not harboring genetic alterations observed in HCC, CLD samples showed hypomethylation of genes such as *HDAC11*, which is highly expressed in immune cells (47), *UBD (FAT10)*, involved in the inflammatory response and immune cell infiltration (47, 48), and *TAGLN2* that controls liver cancer cell motility (47–49). These genes are reported to be upregulated in HCC and in certain cases also to promote tumor progression (50–52). In this way, epigenetic changes during CLD influence the emergence of HCC by epigenetic regulation of gene expression, and could also create a permissive environment for the acquisition of genetic alterations that influence HCC prognosis.

### Transcriptomic Dysregulation in CLD Contributes to Emergence of HCC

Gene expression in CLD patients is associated with HCC outcomes, suggesting that the environment in which HCC develops exerts an influence on tumor development (12, 45, 53, 54). One study investigated gene expression in non-tumoral liver tissues in patients with early-stage HCC and in liver tissue from patients with cirrhosis without HCC (55). They identified an immune-regulatory gene expression signature which is associated with risk of HCC development in cirrhotic patients (median follow up, 10 years). Our group observed increased expression of immune gene sets in CLD but not HCC samples, in line with the shift toward a suppressive immune environment in HCC to allow tumor growth (45, 56). The overexpression of *CTNNB1*, associated with mutations in this gene, occurred in both cirrhotic tissues and tumors and correlated with higher expression of Wnt pathway target genes involved in apoptosis and proliferation, such as *c-Myc* (57). Interestingly, missense mutations in exon-3 of *CTNNB1* do not promote *CTNNB1* overexpression but cause an aberrant activation of the Wnt signaling pathway by preventing phosphorylation and the degradation of ß-catenin. The subset of cases with exon-3 mutations shows a more aggressive phenotype of HCC (58). Of note, *Wnt*/*CTNNB1* mutations correlate with a significant reduction of activated immune cells and immune activation pathways with an increase of M2-type macrophages in HCC patients (58–61). Although often driven by mutation affecting its promoter, TERT overexpression was shown to be associated with macrophage activation in patients with ALD. The study unveiled the cross-talk of TERT with the NF-κB pathway during macrophage polarization (62). Transcriptomic analysis of premalignant cirrhotic nodules and early HCCs also suggests that the MYC transcription signature may play a role in malignant transformation (63). Indeed, E2F and MYC-targets gene sets were also found to be upregulated compared to normal liver in a progressive manner from CLD to HCC (45). In addition, current data indicates a significant link between hepatic inflammation mediators and c-Myc in CLDs. The relationship between c-Myc and aberrant expression of inflammatory mediators play a central role in fibrosis, cirrhosis, and liver cancer. For example, c-Myc upregulates IL-8, IL-10, TNF-α, and TGF-β, while its expression is promoted by IL-1, IL-2, IL-4, TNF-α, and TGF-β (64). Cancer-related pathways, including epithelial-to-mesenchymal-transition (EMT), were identified to be transcriptionally dysregulated in precancerous lesions, independent of genetic alterations (45). The process of EMT in fibrogenic disorders includes phenotypic plasticity, where epithelial cells may dedifferentiate to a mesenchymal-like state in the hostile inflammatory environment, and acquire features such as increased motility (65, 66). Overall, these data highlight how dysregulation of transcriptional programmes may already be activated in CLD and contribute to tumorigenesis.

Tumors occur in a liver microenvironment comprising endothelial, immune and hepatic stellate cells, which contributes to the development of HCC through a complex network of intercellular interactions. Single cell RNA-sequencing (scRNA-seq) enables the study of this heterogeneous environment at the level of individual cells and captures the mutual regulatory mechanisms within a tissue. The transcriptomic profiles of individual cell populations in the liver microenvironment from 4 patients with healthy liver tissues, 3 patients with cirrhosis and 16 cases of HBV-related HCC tissues have been investigated using scRNA-seq and demonstrated the increasing immune cell involvement in each tissue from normal to HCC, relating to inflammatory damage from cirrhosis and the immune response (54). From eight identified genes whose expression in endothelial and stellate cells (liver-resident fibroblasts activated in CLD to support the tumor-promoting microenvironment) correlated with survival in HCC patients (54), four were potential oncogenes with high expression in HCC (*CKS2, HSP90AB1, RPL12, S100A6*) and two were potential tumor-suppressor genes (*CCL14* and *CD5L*) with low expression. The importance of these genes has been corroborated in other studies, for example the silencing of *CKS2* has indeed been shown to inhibit cancer cell proliferation in HCC cells (67). Xing et al. reported that *CD5L* was decreased in liver tissues (54), however CD5L has also been found to be upregulated in HCC and liver cirrhosis (68–72) with a functional involvement in cellular proliferation promotion and antiapoptotic responses by binding to HSPA5 (73). CD5L further emerges as a key player in fibrosis in the setting of CDL as it exerts a protective effect on fibrosis by a combination of mechanisms, including protection from damage, prevention from fibrosis and immune cell infiltration (74). Indeed, CD5L prevents neutrophil and monocyte-derived macrophage infiltration in the inflamed liver (74). Zhang et al. performed scRNAseq on healthy liver, cirrhosis and HCC patients to reveal the landscape of cirrhosis-associated immune cell infiltration in HCC (75). By looking both at differential genes in the B cell developmental trajectory from healthy liver to CLD to HCC and the tumor-associated genes between cirrhotic and malignant hepatocytes, the authors identified enrichment for the humoral immune response pathway and response to oxidative stress (75). In the hepatocytes, they identified key prognostic genes (*FTCD, MARCKSL1, CXCL3, RGS5, KNG1*, and *S100A16*) and JUN transcription factor was suggested to play a role in malignant transformation. In a study investigating tumor-infiltration in HCC at the multi-omics level, *SPP1* was indicated as an immune-related predictor of poor survival and mediator of macrophage-HCC cell interactions (76). Dong et al. used scRNA-seq to compare gene expression between tumor and inflammatory para-tumor tissue, inferring *MLXIPL* as an important transcription factor in the route from normal liver cells to HCC subclones (77). Profiling gene expression in single cell populations in CLD is an expanding area and has the potential to reveal the role of individual cell types in HCC emergence, thus far revealing expression of genes in hepatocytes, and also endothelial cells, stellate cells and B cells, for regulating tumor proliferation and the immune response in the damaged CLD microenvironment.

### Elucidating the Role of CLD in HCC Emergence Through Proteomics and Metabolomics Profiling

Proteomic- and metabolomic-based profiling adds valuable layers of information to the molecular landscape, since proteins and interactions between them are fundamental to cellular behavior. Functional proteomic analysis with regard to three major pathways in the liver (metabolism, complement and coagulation cascades) revealed a division of labor and complementarity among the different liver cell types. Upon mapping liver-disease-related genes to the healthy liver cell types, a high degree of enrichment was noted in the non-parenchymal cells (HSCs, Kupffer cells, liver sinusoidal endothelial cells and immune cells around the hepatocytes (78), highlighting the important regulative role of these cells in immunologic responses and disease development (79). During the progression of chronic liver injury toward fibrosis, the local interaction between Kupffer cells and resident liver macrophages is initiated, triggering the release of cytokines and chemokines (80, 81). The activation and proliferation of HSCs produce Extracellular matrix (ECM) proteins that subsequently form a fibrous scar (82–84). A further comprehensive serum proteomic analysis evaluated changes in protein levels between cirrhosis patients with and without HCC and discovered 21 novel diagnostic biomarkers for HCC enriched for the complement and coagulation cascade pathway (85). Proteins found differentially expressed in cirrhotic samples, FGFR4, TPM4, TPM2, LAALS3BP, and APOA1, were identified as the center of respective protein-protein interaction networks in CLD (86). Pathway enrichment of these networks suggest key roles for these proteins not only in the pathology of cirrhosis but potentially in HCC progression. Aberrant expression of FGFR4 has been reported to contribute to HCC progression (87), and proteins in the corresponding network were enriched for the MAPK cascade, which plays a pivotal role in HCC development due to activation by upstream growth factors (88), whereas APOA1 is prognostic for survival in HCC patients and predictive of early recurrence. Further research is required in following CLD cases to identify predictive biomarkers and to assert the role of CLD in shaping the proteome in HCC development, as large proteogenomics studies have thus far focused on characterizing the tumoral tissue in HCC (89, 90).

Metabolomics provides holistic information on dynamic metabolic responses resulting from perturbations caused by CLD. Metabolites related to ammonia recycling, the urea cycle, and amino acid metabolism were found to discriminate between HCC and cirrhosis. A panel of metabolites including methionine, proline, ornithine, pimelylcarnitine, and octanoylcarnitine demonstrated higher diagnostic accuracy compared to alpha-fetoprotein (AFP) alone and was validated in another cohort (91). Nevertheless, AFP plays an important role in the development and progression of HCC as it activates the PI3K/AKT/mTOR pathway to promote expression of oncogenes such as Ras, Src, and CXCR4. It also leads to immune evasion by inducing apoptosis in antigen-presenting cells and inhibiting proliferation of infiltrating immune cells (92, 93). In an exploratory research, phenylalanine and glutamine were associated with development of HCC in patients with cirrhosis (94) while a larger multicentre study, analyzing a panel of metabolites, identified and phenylalanyl-tryptophan and glycocholate and validated these in an independent cohort. The panel showed a reliable HCC diagnosis in high-risk cirrhosis which, in combination with AFP, improved the panel's sensitivity (95). Metabolomics results indicate the utility of identifying perturbed metabolic pathways for determining biomarkers for cirrhotic patients that are at high risk of developing HCC, but metabolomic profiles of cirrhotic patients differ depending on the underlying etiology (96). There have been a number of studies on the metabolome of cirrhotic patients, as listed by Khan et al. (96), or in studies that discriminate HCC from cirrhotic background (97) which may explain altered pathways during hepato-carcinogenesis. However, there is not a uniform consensus regarding shift of metabolite expression in the emergence of HCC from CLD. This is in part due to the technical limitations including large numbers of metabolites exceeding analysis capabilities, too few metabolites in the database to garner true positive results, or challenges in experimental reproducibility, as well as differences in clinical characteristics in the HCC cohorts between studies.

## Molecular Alterations in Non-resolved Liver Inflammation Predict HCC Recurrence

Recurrence of HCC after therapy or resection represents an important clinical issue as it affects up to 70% of patients within 5 years (1, 2), with risk of late recurrence doubling upon the presence of liver cirrhosis (98). Current evidence suggests that inflammatory signals might increase tumor fitness and reduce expression of “stress ligands” on cancer cells (99–101) thereby allowing the outgrowth of malignant clones (102). This is especially evident in HCC where inflammation-induced tissue injury in CLD, cell death and liver regeneration following resection potentiate tumor outgrowth.

### The Epigenetic Predisposition of CLD Tissues for HCC Recurrence

Recurrent tumors have been shown to harbor distinct genomic profiles and more aggressive phenotypes compared to primary tumors in HCC, suggesting an intracellular reprogramming during tumor recurrence (103). The carcinogenic environment of CLD may foster tumor recurrence through selective pressures for clonal outgrowth as micrometastases, or by formation of multiple *de novo* tumors, with the latter clinically defined as recurrence despite being genetically independent from the primary. Recently, Ding et al. profiled matched initial/recurring tumors and neighboring cirrhotic/fibrotic liver in 133 HCC patients at the genetic and epigenetic (DNA methylation) levels (4). In this study, whole genome sequencing identified a patient with independent multifocal tumors originating from different clones. By contrast, aberrant methylation patterns were maintained from cirrhosis to both tumors, suggesting epigenetic alterations already existed in the CLD tissue. Many early methylation changes linking CLD to HCC were shown to target genes involved in inflammation-associated tumorigenesis, including *SOCS2* hypermethylation and *UBD* hypomethylation, the latter of which was also found hypomethylated in CLD samples by Gallon et al. (45). Interestingly, SOCS2 has a critical role during tumor-immune surveillance (104) and in restraining deleterious immune responses upon severe liver injury (105).

Overall, aberrant methylation patterns and epigenetic alterations in HCC reflect their corresponding cirrhotic/fibrotic livers in the majority of cases, including both initial and recurring tumors. Therefore, these underlying mechanisms that exist in the liver before carcinogenesis highly influence the risk of carcinogenesis from liver diseases to HCC and evidence the relevance of the epigenetic-level field effect.

### Expression of Inflammatory Pathway Genes in CLD Tissues Associate With HCC Recurrence

Persistent liver damage and concomitant non-resolved inflammation is known to alter the genomic landscape of the liver. As discussed above, this specific genetic background subsequently promotes the transformation of hepatocytes into progenitor cells. However, the switch from a normal to a malignant setting has been shown to be marked by the expression of cytokeratin *CK19* in hepatocytes in the presence of inflammation or other stimuli (106). A large number of studies showed that *CK19* also is aberrantly expressed in HCC and gene signatures of this progenitor marker were independent predictors of HCC recurrence after liver transplant (107–111). Despite a lack of association between gene expression profiles in tumor tissues and HCC recurrence in a cohort of 106 patients, a gene-expression signature in the non-tumoral adjacent liver tissues was found to be predictive of late recurrence and overall survival (53). In this study, the genes found to be indicative of poor prognosis were not only enriched for the NF-kB inflammatory response but also for oxidative stress and IL-6 growth signaling pathways. The gene set was further validated in non-tumor tissues with varying etiology, indicating the robustness of the late recurrence signature. Interestingly, death of hepatocytes is known to trigger expression of IL-6 and other growth factors to promote survival and growth of neighboring mutated hepatocytes (112). IL-6 signaling mediated by tumor-associated macrophages was found to promote a cancer stem cell phenotype in HCC, which due to the self-renewal and tumor-initiating capacity of stem cells, is involved in recurrence and metastasis. NF-kB (activated by IL-6) and STAT3 pathways are key orchestrators connecting non-resolving inflammation with HCC, which control the expression of genes involved in cell proliferation and immune functions (3). Transcriptional studies also demonstrate enhanced expression of distinct inflammatory cytokines and chemokines in primary tumors and metastatic lesions (113, 114). In line with this, intrahepatic metastasis in HBV-related HCC was found to be associated with a Th2-dominant cytokine gene expression profile in non-cancerous hepatic tissues from metastatic HCC patients (115). Chronic hepatitis infections result in the production of a cirrhotic liver which can promote the formation of *de novo* tumors, leading to late recurrence. In an effort to identify gene signatures related to HCC recurrence, Okamoto et al. profiled the gene expression of liver tissue of HCV patients and created a prediction score which could stratify the risk of patients of multicentric HCC recurrence (116). To understand gene expression signatures in HCV patients, Tsuchiya and colleagues profiled the tumor and non-tumor tissues of HCV-positive HCC patients. The resulting gene set successfully separated patients with and without HCC recurrence and further analysis revealed that hepatic nuclear factor 4-alpha and interferon gamma were the two main networks involved during HCC recurrence (117). Overall, these results demonstrate that non-tumoral transcriptional profiles can predict HCC recurrence independent of expression in the tumors, pointing toward the tumor microenvironment as being implicated in recurrence after resection. Furthermore, non-resolved inflammation and activation of inflammatory pathways are key in HCC late recurrence and disease progression.

### Proteomics-Level Evidence of Immune Cell Involvement in HCC Recurrence

Tumor-promoting inflammation closely resembles inflammatory mechanisms typically found during immune responses. Hence, the study of the proteome within the inflamed non-tumoral background of HCC provides a framework for analyzing cellular networks and a reference for altered communication associated with pathology. The control of liver inflammation and regeneration is mainly orchestrated by infiltrating inflammatory cells. Upon hepatic injury, resident Kupffer cells, circulating monocytes, macrophages, dendritic cells and neutrophils interact to maintain the liver tissue architecture. Interestingly, studies have shown that upon inflammation triggered by CLDs or surgery, myeloid cells can act in a dual mode, that is by promoting regeneration or, on the contrary, driving inflammatory processes that could aid tumor recurrence (118). In healthy tissues, myeloid cells play a vital role in tissue repair, as well as protective immunity through cytokine secretion and phagocytosis. However, in the underlying environment of non-resolving inflammation in HCC, myeloid cells are shifted toward immunosuppressive and tumor-promoting states such as tumor-associated macrophages and myeloid-derived suppressor cells (119). Between patients with recurrent and non-recurrent HCC, most differentially expressed proteins also belonged to immune system pathways (120). This included LGALS3, which was associated with early recurrence and ability of tumors to suppress the immune system. LGALS3 secreted by the cells of the TME alters immune cell function by inhibiting the expression of T cell receptors and preventing NK cell receptor binding to antigen (121, 122). Interestingly, this lectin was previously found to be expressed in HCC and focal regenerating nodules of cirrhotic tissue, despite being absent in normal hepatocytes (123, 124), possibly representing an early neoplastic event. Autophagy is a fundamental pathway that has multiple effects on immunity and inflammation, by influencing the development, homeostasis and survival of both immune and inflammatory cells. Of note, low concentration of the autophagy-related marker LC3 on HCC and adjacent liver microenvironment can be a significant predictor of both early and late HCC recurrence (125). Interestingly, autophagy proteins in the myeloid cells of the TME contribute to immune suppression of T lymphocytes by effecting LC3-associated phagocytosis. Altered LC3-associated phagocytosis in the myeloid compartment, subsequently induces control of tumor growth by tumor-associated macrophages upon phagocytosis of dying tumor cells (126). An additional class of enzymes involved in HCC recurrence which are typically expressed by the “pro-restorative” macrophage infiltrate (118) are matrix metalloproteinases (MMPs). MMPs are a family of proteolytic enzymes that perform multiple roles in the normal immune response to infection. MMP-9 overexpression by tumor-associated macrophages is associated with higher invasive potential of HCC (118). Interestingly, MMP-9 and MMP-2 positive staining in stromal tissue adjacent to tumors is associated with HCC recurrence in patients with underlying cirrhosis (127). Taken together, these results highlight the key roles of cells in the inflamed tumor microenvironment in cancer development and recurrence.

**Figure 2:**
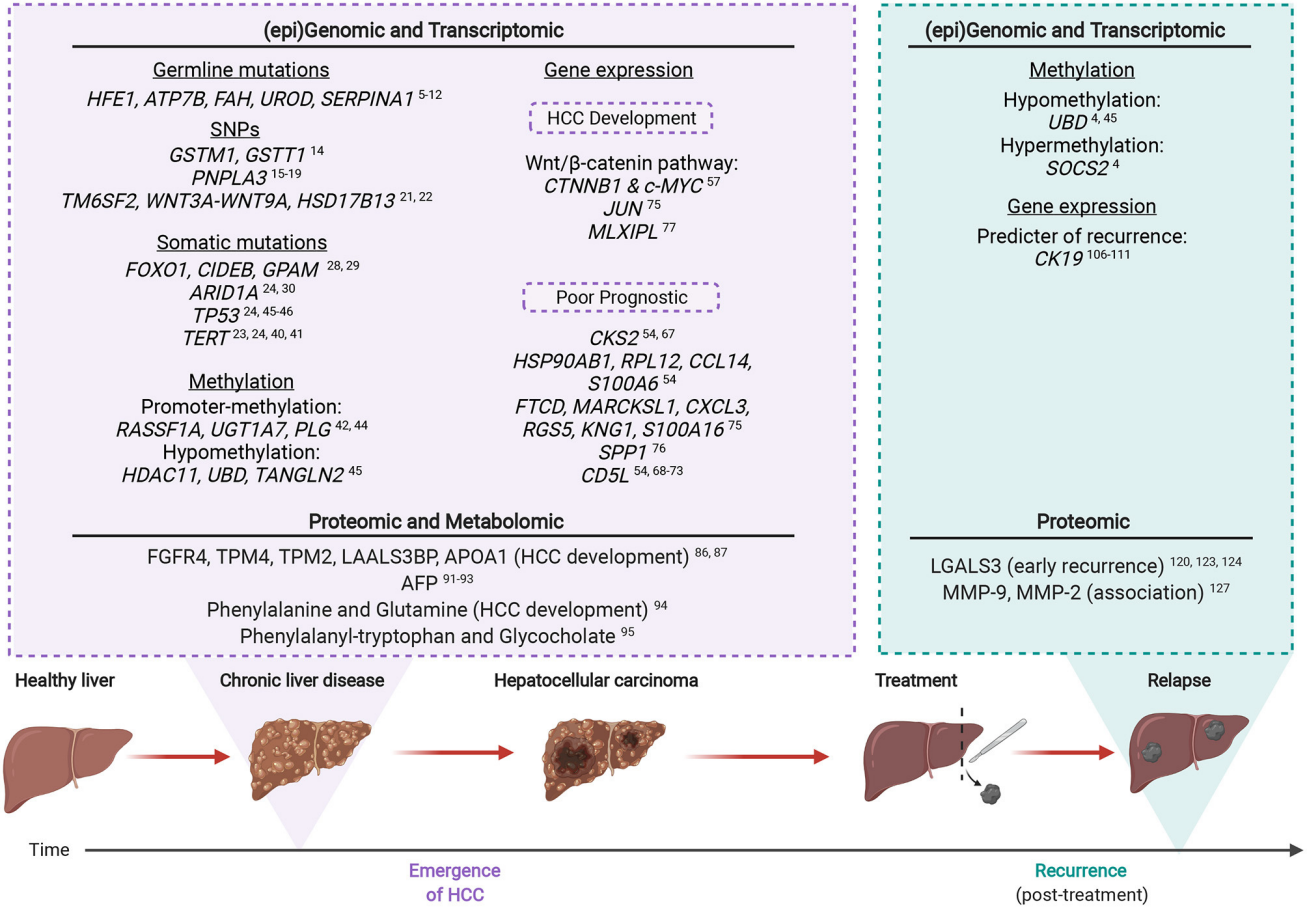
The multi-omic landscape of inflammation-induced liver injury during HCC emergence and recurrence. Most HCCs (>90%) arise on the background of chronic liver inflammation. Molecular alterations analyzed by omic profiling are detectable in the non-tumoral and CLD tissues of inflamed livers prior to HCC (emergence). Upon recurrence, certain genetic mutations or altered protein expression are associated with an increased risk of malignant transformation. Created with www.BioRender.com.

The key omic factors involved in CLD-HCC are summarized in Figure 2, from studies which indicate that HCC emergence and recurrence highly depend on the genomic, transcriptomic, proteomic and metabolomic background of CLD tissues.

## Conclusion

The molecular mechanisms underlying HCC tumorigenesis from a background of CLD are gradually being unveiled through recent sequencing efforts. Omics based studies facilitate the study of global changes in biomolecules in a high throughput manner, and hence are well-poised to reveal the complex changes which lead to HCC emergence and recurrence. Convergent mutations underlie clonal expansion from CLD, indicating selective pressure from the damaged environment, with aberrant methylation patterns from CLD persisting in HCC. In the emergence of HCC from CLD, *TERT* promoter mutations aid hepatocyte fitness and there is evidence of hypomethylation associated with upregulation of tumor-promoting genes, as well as epigenetic silencing of tumor suppressor genes. Current studies support the “epigenetic priming” model, whereby epigenetic changes induced by a prolonged chronic state sensitize cells to genetic mutations and altered transcriptional landscape, triggering tumorigenic progression and recurrence. Further implicated in the emergence of HCC are transcriptional dysregulation of genes involved in the WNT/b-catenin pathway and enrichment of the humoral response pathway, whereas pathways implicated in recurrent HCC include IL-6 growth signaling, NF-kB, IFN-gamma and HNF4a pathways. During both emergence and recurrence, evidence from multi-omic studies predominantly highlights members of the inflammatory response, underlying the key role of a chronically inflamed microenvironment as the molecular link between CLD and HCC. We are only now starting to appreciate the dynamicity, heterogeneity and complexity of the initial steps required to predispose CLD tissues to full blown HCC. Overall, the expansion of ongoing analysis of non-tumoral tissue and CLD will have a major impact in the management of HCC, thus supporting the necessary development and refinement of preventive therapeutic strategies that reflect the molecular dysregulations associated with the CLD background.

## Author Contributions

SP, MC-L, and JG: conceptualization. SZ and GB: literature research and preparation of the first draft of the manuscript. SZ: figure design. SP, CN, MC-L, GH, SS, OK, JG, and GB: critical revision and editing. All authors have read and agreed to the published version of the paper.

## Funding

CN and SP were supported by the Swiss Cancer Research foundation (KFS-4543-08-2018, KFS-4988-02-2020-R, respectively); SZ was supported by AIRC grant number IG 2019 Id.23615; SP was supported by the Professor Dr. Max Cloëtta Foundation; JG was supported by the University of Basel (Research Fund Junior Researchers).

## Conflict of Interest

The authors declare that the research was conducted in the absence of any commercial or financial relationships that could be construed as a potential conflict of interest.

## Publisher's Note

All claims expressed in this article are solely those of the authors and do not necessarily represent those of their affiliated organizations, or those of the publisher, the editors and the reviewers. Any product that may be evaluated in this article, or claim that may be made by its manufacturer, is not guaranteed or endorsed by the publisher.
